# Histologic subtypes are not associated with the presence of sarcopenia in lung cancer

**DOI:** 10.1371/journal.pone.0194626

**Published:** 2018-03-28

**Authors:** Chang Rae Kim, Eun Young Kim, Young Saing Kim, Hee Kyung Ahn, Kun Woo Kim, Yu Mi Jeong, Jeong Ho Kim

**Affiliations:** 1 Department of Radiology, Gachon University Gil Medical Center, Incheon, Republic of Korea; 2 Division of Hematology and Oncology, Department of Internal Medicine, Gachon University Gil Medical Center, Incheon, Republic of Korea; 3 Department of Thoracic and Cardiovascular Surgery, Gachon University Gil Medical Center, Incheon, Republic of Korea; University of South Alabama Mitchell Cancer Institute, UNITED STATES

## Abstract

**Background:**

Sarcopenia is prevalent and a known adverse prognostic effector in lung cancer (LCA). However, the relationship between sarcopenia and histology remains uncertain in LCA.

**Methods:**

Consecutive patients with newly diagnosed LCA (n = 778) between June 2012 and February 2015 were retrospectively reviewed to identify factors associated with sarcopenia. Sarcopenia was defined as CT-determined L3 muscle index (muscle area at L3/height^2^) of < 55 cm^2^/m^2^ for men and < 39 cm^2^/m^2^ for women.

**Results:**

Mean patient age was 67.7 ± 10.8 years, and most (73.1%) were male. The most prevalent histology was adenocarcinoma (44.0%) and 71.6% of patients had stage III or IV disease. The overall prevalence of sarcopenia was 48.2% (60.3% in men, and 15.3% in women). Univariable analysis showed sarcopenia was significantly associated with male gender, age (≥ 65 years), smoking status, lower BMI (< 23 kg/m^2^), advanced stage (III and IV), and high comorbidity score (Charlson index ≥ 3). Furthermore, the prevalence of sarcopenia was higher in squamous cell carcinoma (54.9%) and small cell LCA (56.4%) than in adenocarcinoma (39.8%). Multivariable analyses showed sarcopenia was independently associated with a male gender (odds ratio [OR], 11.13), elderly (OR, 2.02) and low BMI (OR, 6.28), stage IV (OR, 1.98), and high comorbidity (OR, 1.93). However, no significant association was found between histologic subtypes and sarcopenia.

**Conclusions:**

Sarcopenia was found to be significantly associated with old age, male gender, an advanced stage, comorbidities, and low BMI in LCA. However, histology subtype was not an independent factor for the presence of sarcopenia.

## Introduction

Lung cancer is one of the most common malignancies and a leading cause of cancer death in men and women [1]. The disease frequently presents at an advanced stage and its prognosis is poor. TNM stage has the greatest impact on prognosis in lung cancer. Other known poor prognostic factors are poor performance status, a male gender, and weight loss [2, 3]. Weight loss is the classical manifestation of cancer cachexia, is commonly present in lung cancer patients. However, body weight change does not precisely reflect body composition change, and weight loss is uncertain in patients with a large tumor mass or fluid collection, such as, pleural effusion, ascites, or body edema.

Lung cancer is divided into several histologic types and the identification of histologic subtype in lung cancer is important for treatment planning and predicting prognosis. Adenocarcinoma is the most common histologic type, followed by squamous cell carcinoma, and small cell carcinoma. Many driver mutations found in adenocarcinoma, which facilitate individualized treatment and improve outcomes as compared with standard chemotherapy [4].

A relationship between sarcopenia (skeletal muscle mass depletion) and poor prognosis has been identified in several cancers, including lung cancer. Sarcopenia is associated with poor performance status (PS), reduced overall survival, and increased risk of chemotherapy toxicities [5, 6]. Hyper-catabolism caused by tumor metabolism, systemic inflammation, and other tumor-mediated effects have been suggested to be key features of cancer cachexia syndrome, and the pathophysiology may also differ depending on disease stage and cachexia phase [7, 8]. Male gender, an advanced age, the presence of comorbidities, and advanced tumor stage are known to be associated with the prevalence of sarcopenia in cancer patients [9–11].

Systemic inflammation also plays a key role in carcinogenesis, and evidence has accumulated that histologic assessment of infiltration patterns of various inflammatory response components in lung cancer [12]. Cigarette smoking, which promotes widespread inflammatory and mutagenic response that promotes a pro-cancer immune response, is an established risk factor for lung cancer. However, approximately 10% of lung cancers occur in lifelong nonsmokers, indicating that other factors must be etiologically relevant in lung carcinogenesis [12]. In the daily practice, we got impression that lung adenocarcinoma of nonsmokers have lower prevalence of sarcopenia compared with other histology. Most of previous studies evaluated the clinical impact of sarcopenia in advanced stage non-small cell lung cancer (NSCLC) [5]. However, relationship between sarcopenia and histologic subtypes of lung cancer remains uncertain, and these studies were neither designed to detect risk of specific lung cancer types. The knowledge of these associations might provide better understanding of the mechanism responsible for sarcopenia in lung cancer.

Accordingly, the purpose of this study was to determine whether a relationship exists between the prevalence of sarcopenia and histologic subtypes in lung cancer using CT images taken at time of initial diagnosis.

## Materials and methods

### Patients

The radiology database and medical records system at Gachon University Gil Medical Center (Incheon, Korea) were reviewed respectively. Seven hundred and seventy eight consecutive patients with newly diagnosed lung cancer between June 2012 and February 2015 were enrolled.

Height and weight were measured and functional status was recorded at first visit to our oncology department. Body mass index (BMI) was defined as weight divided by height squared (kg/m^2^), and BMI values were categorized as underweight (<18.5 kg/m^2^), normal (18.5–22.9 kg/m^2^), overweight (23.0–24.9 kg/m^2^), or obese (≥25 kg/m^2^).

Lung cancer histology was classified as adenocarcinoma, squamous cell carcinoma, small cell lung cancer, and others (large cell carcinoma, adenosquamous carcinoma, and non-small cell lung cancer other than specified). Tumor stages were determined as described in the American Joint Commission on Cancer (AJCC) Staging Manual (7^th^ edition) [13].

Ethics approval for this study was granted by the Gil Medical Center Institutional Review Board (approval number: GBIRB-2017-218).

### PET/CT

All patients fasted for at least 6 hours before PET/CT to ensure a normal blood glucose level. About 60 minutes after the intravenous administration of 370 MBq (10 mCi) of FDG, imaging was performed using an integrated PET/CT device (Siemens Medical Systems, Erlangen, Germany), equipped with lutetium oxyorthosilicate crystal PET detectors, and six slices of CT detectors.

Integrated CT imaging was performed from the head to the pelvic floor for anatomical localization and attenuation correction without contrast administration using the following parameters: 130 kVp, 110 mAs, 2-mm pitch, 1-second tube rotation, and a slice thickness of 5 mm, which matched the slice thickness of PET images. Subsequent PET scanning was performed using five to eight table positions to provide adequate coverage from head to pelvic floor. CT data were used for attenuation correction and PET image data were reconstructed using an ordered set expectation maximization algorithm.

### Image analysis

Baseline CT images obtained at time of diagnosis were retrospectively analyzed by a subspecialty-trained chest radiologist. The third lumbar vertebra (L3) was selected as a landmark because the cross-sectional area of tissues in this region provide an established means of estimating total body tissue quantities in the general population with reported Pearson’s correlation coefficients ranging from 0.71 to 0.92 [14]. Two consecutive CT images extending from L3 in the inferior direction were assessed.

**Fig 1:**
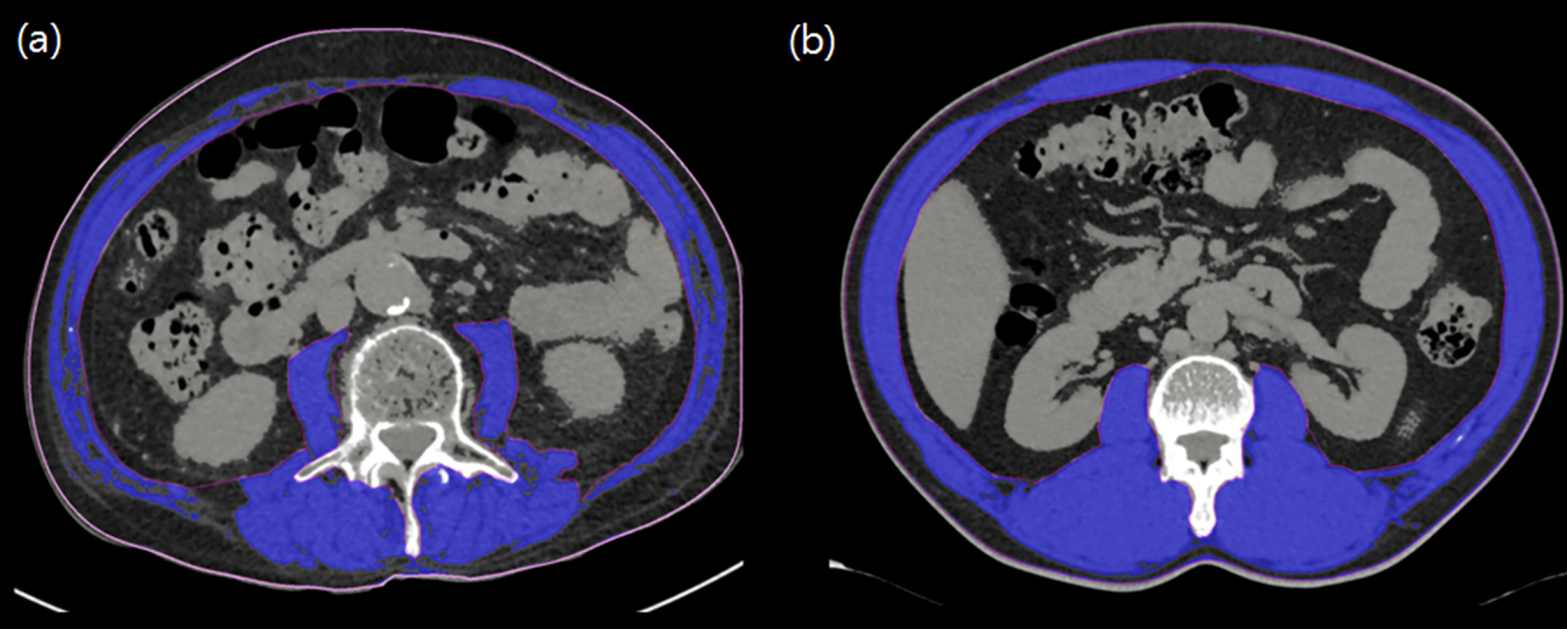
Single cross-sectional area of skeletal muscle at the third lumbar vertebrae in patients with lung cancer. (A) In this 71-year-old male patient (stage III, squamous cell carcinoma, Charlson comorbidity score of 5, BMI 22.2 kg/m^2^) with sarcopenia, the CT-measured L3 muscle (blue color) index was 33.0 cm^2^/m^2^. (B) In this 54-year-old male patient (stage I, adenocarcinoma, Charlson comorbidity score of 3, BMI 29.8 kg/m^2^) without sarcopenia, the CT-measured L3 muscle (blue color) index was 86.9 cm^2^/m^2^.

Body composition analyses were performed using commercially available software (Terarecon 3.4.2.11, San Mateo, CA). Tissue cross-sectional areas (cm^2^) of muscle masses in slices were computed automatically by summing appropriate pixels (the CT Hounsfield unit (HU) range used to delineate skeletal muscle was −29 to 150 HU) (Fig 1.). After applying threshold methods using a predefined HU threshold set for each slice, boundaries between different tissues were corrected manually when necessary. L3 muscle index (cm^2^/m^2^) was defined as the cross-sectional area of muscle at the L3 level normalized for stature, as is conventional for BMI.

### Definition of sarcopenia

Sarcopenia was defined as a L3 muscle index of < 55 cm^2^/m^2^ for men and < 39 cm^2^/m^2^ for women, as proposed by international consensus for cancer cachexia [15].

### Statistical analysis

Descriptive statistics are reported as proportions or means with standard deviations. For categorical variables, comparisons between subjects with or without sarcopenia were performed using Pearson’s χ2 test. Continuous variables were compared using the Student’s t test. Univariable and multivariable regression analysis were used to explore associations between sarcopenia and patient demographic and disease-related factors, which included age, gender, BMI, smoking status, histologic types, cancer stage, and Charlson comorbidity index. In order to identify important predictors for the multivariable model, the enter method with F-statistic were applied when candidate variables had a *p* value of < 0.05 by univariable analysis. The statistical analysis was performed using SPSS for Windows ver. 19.0 (SPSS Inc., Chicago, IL, USA), and statistical significance was accepted for *p* values < 0.05.

## Results

### Characteristics of the study population

A total of 778 consecutive patients were included in this study, and 569 patients (73.1%) were male (Table 1). Overall mean age of the study subjects was 67.7 ± 10.8 years. The most prevalent histology was adenocarcinoma (n = 342, 44.0%), followed by squamous cell carcinoma (n = 193, 24.8%), and small cell lung cancer (n = 133, 17.1%). Most of patients had stage III (n = 180, 23.1%) or IV (n = 377, 48.5%) disease.

**Table 1:** Comparison of the characteristics of lung cancer patients with or without sarcopenia.

Variables	Total (n = 778)	Sarcopenia (n = 375)	No sarcopenia (n = 403)	*p* value
**Sex**				
Male	569 (73.1%)	343 (91.5%)	226 (56.1%)	< 0.001*
Female	209 (26.9%)	32 (8.5%)	177 (43.9%)	
**Age (years)**				
Mean (SD)	67.7 (10.8)	69.9 (10.9)	65.7 (10.2)	< 0.001^†^
Elderly, **≥** 65 yrs	499 (64.1%)	272 (72.5%)	227 (56.3%)	
**Cancer Histology**				
Adenocarcinoma	342 (44.0%)	134 (35.7%)	208 (51.6%)	< 0.001*
Squamous cell carcinoma	193 (24.8%)	106 (28.3%)	87 (21.6%)	
Small cell lung cancer	133 (17.1%)	75 (20.0%)	58 (14.4%)	
Others^¶^	110 (14.1%)	60 (16.0%)	50 (12.4%)	
**Cancer Stage (TNM)**				
I	132 (17.0%)	40 (10.7%)	92 (22.8%)	< 0.001*
II	89 (11.4%)	37 (9.9%)	52 (12.9%)	
III	180 (23.1%)	89 (23.7%)	91 (22.6%)	
IV	377 (48.5%)	209 (55.7%)	168 (41.7%)	
**Charlson comorbidity index**				
0–2	662 (85.1%)	309 (82.4%)	353 (87.6%)	.042*
≥3	116 (14.9%)	66 (17.6%)	50 (12.4%)	
**Smoking Status**				
Current or ex-smoker	575 (73.9%)	328 (87.5%)	247 (61.3%)	< 0.001*
Never-smoker	203 (26.1%)	47 (12.5%)	156 (38.7%)	
**BMI (kg/m**^**2**^**)**				
Mean (SD)	22.8 (3.5)	21.3 (2.9)	24.2 (3.4)	< 0.001^†^
Underweight	84 (10.8%)	66 (17.6%)	18 (4.5%)	
Normal	336 (43.2%)	208 (55.5%)	128 (31.8%)	
Overweight	162 (20.8%)	64 (17.1%)	98 (24.3%)	
Obese	196 (25.2%)	37 (9.9%)	159 (39.5%)	
**L3 muscle index (kg/m**^**2**^**)**				
Mean (SD)	51.3 (9.9)	45.7 (6.5)	56.6 (9.6)	< 0.001^†^

*Pearson’s chi-squared test.

^†^Student’s t test.

^¶^includes adenosquamous cancer, large cell carcinoma, and non-small cell lung cancer other than specified

The driver genetic abnormalities were investigated in non-small cell lung cancer (NSCLC); excluding patients whose information of EGFR (n = 62) and ALK (n = 54) was unavailable, epidermal growth factor receptor (EGFR) mutations was found in 32.6% and anaplastic lymphoma kinase (ALK) gene rearrangements was found in 4.7% in adenocarcinoma patients.

Overall mean BMI was 22.8 ± 3.5 kg/m^2^, and male patients had a significantly lower BMI (mean BMI, 22.6 ± 3.3 kg/m^2^ for male vs. 23.3 ± 4.0 kg/m^2^ for female, *p* = 0.026) and a significantly higher L3 muscle index (mean L3 muscle index, 53.0 ± 10.1 cm^2^/m^2^ for male, 47.0 ± 7.9 cm^2^/m^2^ for female, *p* < 0.001).

### Comparison between sarcopenic and non-sarcopenic patients

Of the total 778 patients, sarcopenia was present in 375 (48.2%) as determined by CT taken at time of diagnosis (Table 1). Sarcopenia was more prevalent in men (60.3% for males and 15.3% for females). Sarcopenic patients were significantly older (mean age, 69.9 ± 10.9 years vs. 65.7 ± 10.2 years, *p* < 0.001) and had lower BMIs (mean BMI, 21.3 ± 2.9 kg/m^2^ vs. 24.2 ± 3.4 kg/m^2^, *p* < 0.001). In addition, the prevalence of sarcopenia showed a significant increasing trend with advanced stage (30.3% for stage I, 41.6% for stage II, 49.4% for stage III, and 55.4% for stage IV, *p* < 0.001 as determined using the linear-by-linear association test). Furthermore, the prevalence of sarcopenia was lower for adenocarcinoma (39.8%) than for squamous cell carcinoma (54.9%) or small cell carcinoma (56.4%).

A high comorbidity level (Charlson comorbidity index ≥ 3) and a smoking history (current or ex-smoker) were more common in sarcopenic patients (*p* = 0.042 and *p* < 0.001, respectively).

### Factors associated with the prevalence of sarcopenia

Univariable and multivariable logistic regression analyses results are summarized in Table 2. Univariable analysis showed a non-adenocarcinoma histology, age (age ≥ 65 years), male gender, advanced stage (stage III or IV), high comorbidity (Charlson comorbidity index ≥ 3), low BMI (< 23 kg/m^2^), and a smoking history were associated with a higher prevalence of sarcopenia.

**Table 2:** Results of univariable and multivariable analyses of factors associated with the presence of sarcopenia in lung cancer patients.

	Univariable odds ratio (95% CI)	*p* value	Multivariable odds ratio (95% CI)	*p* value
**Age, years**				
Non-elderly	1.00 (reference)		1.00 (reference)	
Elderly (≥ 65yrs)	2.05 (1.52–2.77)	<0.001	2.02 (1.39–2.93)	< 0.001
**Gender**				
Female	1.00 (reference)		1.00 (reference)	
Male	8.34 (5.56–12.68)	< 0.001	11.13 (6.05–20.47)	< 0.001
**Cancer types**				
Adenocarcinoma	1.00 (reference)		1.00 (reference)	
Squamous cell carcinoma	1.89 (1.32–2.70)	< 0.001	0.93 (0.59–1.47)	0.761
Small cell lung cancer	2.01 (1.34–3.01)	0.001	1.10 (0.66–1.84)	0.711
Others^¶^	1.86 (1.21–2.87)	0.005	1.06 (0.62–1.82)	0.827
**Cancer Stage (TNM)**				
I	1.00 (reference)		1.00 (reference)	
II	1.64 (0.93–2.87)	0.086	0.90 (0.46–1.79)	0.770
III	2.25 (1.40–3.61)	0.001	1.40 (0.79–2.49)	0.250
IV	2.86 (1.87–4.37)	< 0.001	1.98 (1.16–3.37)	0.012
**Charlson comorbidity index**				
0–2	1.00 (reference)		1.00 (reference)	
≥3	1.51 (1.01–2.25)	0.043	1.93 (1.19–3.13)	0.008
**Body mass index (kg/m**^**2**^**)**				
≥ 23	1.00 (reference)		1.00 (reference)	
< 23	4.78 (3.52–6.48)	< 0.001	6.28 (4.37–9.02)	< 0.001
**Smoking history**				
Never-smoker	1.00 (reference)		1.00 (reference)	
Current or ex-smoker	4.41 (3.06–6.35)	< 0.001	1.00 (0.56–1.80)	0.995

However, multivariable analysis failed to identify a significant association between histologic subtypes and the prevalence of sarcopenia. The independent factors found to be related to sarcopenia were; male sex (odds ratio [OR], 11.13; 95% confidence interval [CI], 6.05 to 20.47), old age (OR, 2.02; 95% CI, 1.39 to 2.93), a low BMI (OR, 6.28; 95% CI, 4.37 to 9.02), stage IV (OR, 1.98; 95% CI, 1.16 to 3.37), and a high comorbidity score (OR, 1.93; 95% CI, 1.19 to 3.13).

## Discussion

Cancer cachexia is a multi-factorial syndrome defined as an ongoing wasting of skeletal muscle mass refractory to conventional nutritional support and that leads to progressive functional impairment [15]. Its pathophysiology is characterized by negative protein and energy balances driven by a variable combination of reduced food intake and abnormal metabolism [16].

Sarcopenia is a prevalent condition and is associated with functional impairment, increased risk of chemotherapy-related toxicities, and reduced survival in lung cancer [5, 17], and its prognostic significance in lung cancer emphasizes the need for identifying its presence and for early therapeutic interventions aimed at increasing skeletal muscle mass.

The most common cancer types associated with sarcopenia are lung, genitourinary, and gastric cancer [18]. Although lung cancer has been reported to exhibit a prevalence of sarcopenia, previous studies included lung cancer patients with locally advanced or recurrent (stages III or IV) NSCLC and patients with early stage disease were not included [5, 19]. Furthermore, no report has been issued on the relationship between lung cancer histology and the prevalence of sarcopenia. We considered knowledge of an association between tumor-related factors and sarcopenia might provide better understanding of the underlying mechanism of cancer cachexia in lung cancer, enable the early detection of patients at risk of sarcopenia, and facilitate the adoption of effective therapeutic interventions to increase skeletal muscle mass.

In the present study, we evaluated the prevalence of CT-determined sarcopenia at time of diagnosis in 778 consecutive lung cancer patients. The overall prevalence of sarcopenia in our cohort was 48.2%, and sarcopenia was more prevalent in male and advanced stage patients. Furthermore, the prevalence of sarcopenia was lower in adenocarcinoma than in squamous cell carcinoma or small cell carcinoma.

The diagnosis of lung cancer histology is important for treatment decision-making and for predicting prognosis. SCLC is highly responsive to initial chemotherapy and radiotherapy, but relapse is common and the prognosis is poor [20, 21]. For resectable NSCLC, surgical resection offers the best opportunity for long term survival and cure. On the other hand, in the advanced disease setting, the sub-classification of NSCLC is important for treatment decisions, because driver genetic abnormalities, such as, EGFR mutations and ALK gene rearrangements, have only been validated for a non-squamous histology [22]. Under the impression adenocarcinoma has a lower prevalence of sarcopenia than other histology subtypes, we sought to identify factors associated with sarcopenia in lung cancer patient. Multivariable analysis showed sarcopenia was significantly associated with a male gender, old age (≥ 65 years), low BMI (< 23 kg/m^2^), and a high comorbidity score (Charlson comorbidity index ≥ 3). Furthermore, our findings confirmed a higher prevalence of sarcopenia in stage IV than in stage I lung cancer. Although the prevalence of sarcopenia was found to be significantly lower for adenocarcinoma than for the other histologic types by univariable analysis, this significance was not confirmed by multivariable analysis.

Despite recent interest in the clinical implications of sarcopenia in cancer patients, data on the actual prevalence of sarcopenia across cancer types and stages is limited, presumably because the definition of sarcopenia remains controversial and multiple definitions have been used in the literature [5, 6]. Most published studies have used arbitrary study-specific cutoff values and different imaging modalities, such as, bioelectrical impedance analysis (BIA), dual energy X-ray absorptiometry (DEXA), or CT to determine the presence of sarcopenia. However, CT is the current gold standard modality in body composition research as it precisely differentiates fat and other soft tissues from skeletal muscles. In this study, we used CT images obtained from initial PET/CT scans, which are routinely conducted in oncology patients, and provided a means of precisely quantifying skeletal muscle masses by secondary analysis without additional costs or radiation exposure. Furthermore, sarcopenia in the present study was defined using standard cutoff values of CT-determined L3 muscle index as proposed in a recently published consensus definition of cancer cachexia [15].

Several limitations of the present study should be mentioned. First, the information such driver genetic mutation cannot be obtained for all patients since this study was conducted in retrospective manner. Further in-vitro and in-vivo study would be needed for the verification of the relationship between histologic subtype, driver genetic mutation and the presence of sarcopenia in lung cancer. Second, the patients’ number was small because this study was performed at a single institution.

## Conclusion

In summary, we estimated the prevalence of sarcopenia in newly diagnosed, consecutive lung cancer patients and analyzed factors associated with CT-determined sarcopenia. The prevalence of sarcopenia was found to be independently associated with age (≥65 years), male gender, high comorbidities Charlson comorbidity index ≥ 3), low BMI (< 23 kg/m^2^), and advanced stage (stage IV). However, in this large-scale retrospective study, multivariable analysis showed lung cancer histology is not independently associated with the prevalence of sarcopenia.
